# iLncDA-RSN: identification of lncRNA-disease associations based on reliable similarity networks

**DOI:** 10.3389/fgene.2023.1249171

**Published:** 2023-08-08

**Authors:** Yahan Li, Mingrui Zhang, Junliang Shang, Feng Li, Qianqian Ren, Jin-Xing Liu

**Affiliations:** School of Computer Science, Qufu Normal University, Rizhao, China

**Keywords:** lncRNA-disease association, reliable similarity network, random forest, random walk with restart, elastic net

## Abstract

Identification of disease-associated long non-coding RNAs (lncRNAs) is crucial for unveiling the underlying genetic mechanisms of complex diseases. Multiple types of similarity networks of lncRNAs (or diseases) can complementary and comprehensively characterize their similarities. Hence, in this study, we presented a computational model iLncDA-RSN based on reliable similarity networks for identifying potential lncRNA-disease associations (LDAs). Specifically, for constructing reliable similarity networks of lncRNAs and diseases, miRNA heuristic information with lncRNAs and diseases is firstly introduced to construct their respective Jaccard similarity networks; then Gaussian interaction profile (GIP) kernel similarity networks and Jaccard similarity networks of lncRNAs and diseases are provided based on the lncRNA-disease association network; a random walk with restart strategy is finally applied on Jaccard similarity networks, GIP kernel similarity networks, as well as lncRNA functional similarity network and disease semantic similarity network to construct reliable similarity networks. Depending on the lncRNA-disease association network and the reliable similarity networks, feature vectors of lncRNA-disease pairs are integrated from lncRNA and disease perspectives respectively, and then dimensionality reduced by the elastic net. Two random forests are at last used together on different lncRNA-disease association feature sets to identify potential LDAs. The iLncDA-RSN is evaluated by five-fold cross-validation to analyse its prediction performance, results of which show that the iLncDA-RSN outperforms the compared models. Furthermore, case studies of different complex diseases demonstrate the effectiveness of the iLncDA-RSN in identifying potential LDAs.

## 1 Introduction

Evidences from many studies suggest that the complex process of cancer development is regulated not only by protein-coding RNAs but also by long non-coding RNAs (lncRNAs), a class of RNAs larger than 200 bp with no coding potential (Schmitt and Chang, 2016; Wong et al., 2018). With in-depth research on associations between diseases and lncRNAs, lots of lncRNAs have been identified to have oncogenic potential and cancer-suppressive effects (Taniue and Akimitsu, 2021). For example, the expression of lncRNA HOTAIR is significantly associated with poor prognosis in lung, colon and primary breast cancers, which implies that it may be used as biomarkers for cancer diagnosis and prognosis, as well as potential treatment targets for various cancer types (Gupta et al., 2010; Aprile et al., 2020b). The lncRNA NORAD facilitates cancer development, whose expression is upregulated and associated with poor prognosis in several cancers, including bladder, squamous cell, breast, colorectal, esophageal, and pancreatic cancers (Li et al., 2017; Li et al., 2018; Tan et al., 2019; Zhou et al., 2019; Aprile et al., 2020a; Soghli et al., 2021). Besides, some lncRNAs play essential roles in the regulation of tumor suppressor functions. For instance, the expression of lncRNA GAS5 is negatively related to tumor size, metastasis and stage in prostate, pancreatic, colon, bladder and breast cancer (Goustin et al., 2019). Therefore, identifying potential disease-associated lncRNAs will be helpful for understanding the disease pathogenesis, and facilitating the diagnosis and therapeutics of complex diseases.

Nowadays, more and more biologically validated lncRNA-disease associations (LDAs) are reported, which make it possible to use computational models to predict potential LDAs (Chen and Yan, 2013). Introduced a semi-supervised framework LRLSLDA to identify LDAs, in which the hypothesis of similar diseases normally being associated with similar lncRNAs was proposed. Based on this hypothesis, a series of computational models were developed, which can be mainly divided into three categories, including matrix decomposition, random walk, and machine learning. For the matrix decomposition category (Lu et al., 2018), proposed the SIMCLDA, which uses the principal feature vectors in the constructed feature matrices to complement the association matrix based on an inductive matrix complementation framework. (Wang et al., 2021) regarded as the association prediction problem as the problem of recommendation system, and presented the LDGRNMF to employ graph-regularized nonnegative matrix decomposition to identify potential LDAs. (Liu et al., 2021) proposed the DSCMF to predict potential LDAs, which deals with the sparsity by adding $l_{2,1}-norm$ to the collaboration matrix decomposition. For the random walk category, (Sun et al., 2014) developed the RWRlncD by applying random walk with restart (RWR) strategy to the functional similarity network of lncRNAs to predict potential LDAs. (Gu et al., 2017) presented the GrwLDA, which belongs to the semi-supervised learning method, and can be used for capturing potential associations with isolated diseases or lncRNAs having no known associations. (Li et al., 2021) presented the LRWHLDA based on the local random walk strategy, which can identify potential LDAs in the absence of known LDAs. For the machine learning category, (Zeng et al., 2020) proposed the SDLDA, which uses deep learning and singular value decomposition (SVD) to extract nonlinear and linear features of diseases and lncRNAs, and then trains the model to predict potential LDAs. (Zhu et al., 2021) presented the IPCARF to identify LDAs, which integrates the disease semantic similarity, lncRNA functional similarity and the Gaussian interaction profile (GIP) kernel similarity to obtain feature vectors of lncRNA-disease pairs, and employs incremental principal component analysis to obtain the optimal subspace, which are then trained by the random forest to predict potential LDAs.

Although these models show promising results, there are still several limitations. For instance, some of them only used one type of similarity network of lncRNAs or diseases, which only describe their biological characteristics in a single perspective. It is confirmed that multiple types of similarity networks of lncRNAs (or diseases) can complementary and comprehensively characterize their similarities. However, it is a challenge to properly integrate them without bringing in redundancy and noises. Besides, heuristic information or priori knowledge of other biomolecules that associated with lncRNAs and/or diseases should be considered in the model to fully identifying potential LDAs. Taking the lncRNA-miRNA interaction as an example, the lncRNA MALAT1 has been proven to act as a sponge for miRNA miR-129-5p promoting the development of triple-negative breast cancer (Volovat et al., 2020).

In this study, we proposed a computational model, namely, iLncDA-RSN in short, to identify potential LDAs, which based on reliable similarity networks for integrating multiple types of similarity networks and utilizing miRNA heuristic information. Specifically, for constructing reliable similarity networks of lncRNAs and diseases, miRNA heuristic information with lncRNAs and diseases is firstly introduced to construct their respective Jaccard similarity networks; then GIP kernel similarity networks and Jaccard similarity networks of lncRNAs and diseases are provided based on the lncRNA-disease association network; a random walk with restart strategy is finally applied on Jaccard similarity networks, GIP kernel similarity networks, as well as lncRNA functional similarity network and disease semantic similarity network to construct reliable similarity networks. Depending on the lncRNA-disease association network and the reliable similarity networks, feature vectors of lncRNA-disease pairs are integrated from lncRNA and disease perspectives respectively, and then dimensionality reduced by the elastic net. Two random forests are at last used together on different lncRNA-disease association feature sets to identify potential LDAs. The iLncDA-RSN is evaluated by five-fold cross-validation to analyse its prediction performance, results of which show that the iLncDA-RSN outperforms the compared models. Furthermore, case studies of different complex diseases demonstrate the effectiveness of the iLncDA-RSN in identifying potential LDAs.

## 2 Methods

### 2.1 Disease similarity networks

#### 2.1.1 Disease semantic similarity network and GIP kernel similarity network

The disease semantic similarity network is constructed using disease ontology information containing multiple directed acyclic graphs (Schriml et al., 2012). The disease D can be described as the directed acyclic graph $DAG\left(D\right)=\left(D,T\left(D\right),E\left(D\right)\right)$ , where $T\left(D\right)$ is the set of disease nodes including its ancestors and itself, and $E\left(D\right)$ is the set of edges associated with $T\left(D\right)$ . The disease semantic value $DV\left(D\right)$ of the disease D is defined as,

$$DV\left(D\right)=\sum_{t\in T\left(D\right)}D_{D}\left(t\right)$$ (1)

where $D_{D}\left(t\right)$ represents the semantic contribution of the ancestor disease t to the disease D , and can be written as,

$$D_{D}\left(t\right)=\left\{\begin{array}{cc} 1, & t=D\\ \max\left\{\Delta \times D_{D}\left(t^{\prime }\right)\left|t^{\prime }\in children\;of\;t\right.\right\}, & t\neq D \end{array}\right.$$ (2)

where the semantic contribution factor Δ is usually set to 0.5 (Wang et al., 2010). Based on the assumption of more similar two diseases sharing more directed acyclic graphs, the semantic similarity value $DSS\left(d_{i},d_{j}\right)$ between diseases $d_{i}$ and $d_{j}$ is defined as,

$$DSS\left(d_{i},d_{j}\right)=\frac{\sum_{t\in T\left(d_{i}\right)\cap T\left(d_{j}\right)}\left(D_{d_{i}}\left(t\right)+D_{d_{j}}\left(t\right)\right)}{DV\left(d_{i}\right)+DV\left(d_{j}\right)}$$ (3)

Under the assumption that diseases with similar phenotypes tend to be more associated with similar lncRNAs, and *vice versa*, based on the lncRNA-disease association network, the GIP kernel similarity value $GIPD\left(d_{i},d_{j}\right)$ between diseases $d_{i}$ and $d_{j}$ is computed by,

$$GIPD\left(d_{i},d_{j}\right)=\exp\left(-\gamma_{d}{\left\|IP\left(d_{i}\right)-IP\left(d_{j}\right)\right\|}^{2}\right)$$ (4)

$$\gamma_{d}=1/\left(\sum_{k=1}^{n_{d}}{\left\|IP\left(d_{k}\right)\right\|}^{2}\right)$$ (5)

where $IP\left(d_{i}\right)$ represents the vector of disease $d_{i}$ in the lncRNA-disease association matrix, $\gamma_{d}$ controls the kernel bandwidth, and $n_{d}$ is the number of diseases. Since some diseases have the semantic similarity values and others not, in order to complement these missing values, we integrated the semantic similarity and the GIP kernel similarity together as the disease integrated similarity, which is defined as,

$$SD\left(d_{i},d_{j}\right)=\left\{\begin{array}{cc} \frac{DSS\left(d_{i},d_{j}\right)+GIPD\left(d_{i},d_{j}\right)}{2} & DSS\left(d_{i},d_{j}\right)\;exists\\ GIPD\left(d_{i},d_{j}\right) & otherwise \end{array}\right.$$ (6)

where $SD\left(d_{i},d_{j}\right)$ is the disease integrated similarity value between diseases $d_{i}$ and $d_{j}$ .

#### 2.1.2 Disease Jaccard similarity network based on the lncRNA-disease association network

Jaccard similarity is a common statistic used to describe the degree of similarity between two groups of items and has been widely applied in the calculation of biological data (Luo et al., 2017; Zhou et al., 2021). Based on the lncRNA-disease association network, the disease Jaccard similarity value $JD_{LD}\left(d_{i},d_{j}\right)$ between diseases $d_{i}$ and $d_{j}$ is described as,

$$JD_{LD}\left(d_{i},d_{j}\right)=\frac{\left|IP_{LD}\left(d_{i}\right)\cap IP_{LD}\left(d_{j}\right)\right|}{\left|IP_{LD}\left(d_{i}\right)\cup IP_{LD}\left(d_{j}\right)\right|}$$ (7)

where $IP_{LD}\left(d_{i}\right)$ is the vector of disease $d_{i}$ in the lncRNA-disease association matrix, the same as the representation of $IP\left(d_{i}\right)$ .

#### 2.1.3 Disease Jaccard similarity network based on the miRNA-disease association network

It is believed that heuristic information of other biomolecules that associated with diseases can help to provide supplementary prior knowledge for accurately identifying potential LDAs. In this study, miRNA-disease association network is introduced for calculating the disease Jaccard similarity value $JD_{MD}\left(d_{i},d_{j}\right)$ between diseases $d_{i}$ and $d_{j}$ , which is defined as,

$$JD_{MD}\left(d_{i},d_{j}\right)=\frac{\left|IP_{MD}\left(d_{i}\right)\cap IP_{MD}\left(d_{j}\right)\right|}{\left|IP_{MD}\left(d_{i}\right)\cup IP_{MD}\left(d_{j}\right)\right|}$$ (8)

where $IP_{MD}\left(d_{i}\right)$ is the vector of disease $d_{i}$ in the miRNA-disease association network.

### 2.2 LncRNA similarity networks

#### 2.2.1 LncRNA functional similarity network and GIP kernel similarity network

The computation of functional similarity between two lncRNAs is based on the assumption that lncRNAs with shared functions are more probable correlated with diseases with similar phenotypes (Chen et al., 2015). Suppose the disease set $D_{1}=\left\{d_{11},d_{12},\cdots ,d_{1m}\right\}$ is associated with the lncRNA $l_{i}$ , and the disease set $D_{2}=\left\{d_{21},d_{22},\cdots ,d_{2n}\right\}$ is associated with the lncRNA $l_{j}$ , where m and n are disease numbers in their respective sets, the semantic similarity value $DSS\left(d,D_{2}\right)$ between the disease $d\in D_{1}$ and the disease set $D_{2}$ is defined as,

$$DSS\left(d,D_{2}\right)=\max_{1\leq i\leq n,d_{i}\in D_{2}}\left(DSS\left(d,d_{i}\right)\right)$$ (9)

According to the definition of the semantic similarity value $DSS\left(d,D_{2}\right)$ , the lncRNA functional similarity value $LFS\left(l_{i},l_{j}\right)$ between lncRNAs $l_{i}$ and $l_{j}$ is defined as,

$$LFS\left(l_{i},l_{j}\right)=\frac{\sum_{1\leq i\leq m}DSS\left(d_{1i},D_{2}\right)+\sum_{1\leq j\leq n}DSS\left(d_{2j},D_{1}\right)}{m+n}$$ (10)

Similar with the computational process of the GIP kernel similarity value between two diseases, based on the lncRNA-disease association network, the GIP kernel similarity value $GIPL\left(l_{i},l_{j}\right)$ between lncRNAs $l_{i}$ and $l_{j}$ is defined as (Chen and Yan, 2013),

$$GIPL\left(l_{i},l_{j}\right)=\exp\left(-\gamma_{l}{\left\|IP\left(l_{i}\right)-IP\left(l_{j}\right)\right\|}^{2}\right)$$ (11)

$$\gamma_{l}=1/\left(\sum_{k=1}^{n_{l}}{\left\|IP\left(l_{k}\right)\right\|}^{2}\right)$$ (12)

where $IP\left(l_{i}\right)$ represents the vector of lncRNAs $l_{j}$ in the lncRNA-disease association matrix, $\gamma_{l}$ controls the kernel bandwidth, and $n_{l}$ is the number of lncRNAs. Since some lncRNAs have the functional similarity values and others not, in order to complement these missing values, we integrated the functional similarity and the GIP kernel similarity together as the lncRNA integrated similarity, which is defined as,

$$SL\left(l_{i},l_{j}\right)=\left\{\begin{array}{cc} \frac{LFS\left(l_{i},l_{j}\right)+GIPL\left(l_{i},l_{j}\right)}{2} & LFS\left(l_{i},l_{j}\right)\;exists\\ GIPL\left(l_{i},l_{j}\right) & otherwise \end{array}\right.$$ (13)

where $SL\left(l_{i},l_{j}\right)$ is the lncRNA integrated similarity value between lncRNAs $l_{i}$ and $l_{j}$ .

#### 2.2.2 LncRNA Jaccard similarity network based on the lncRNA-disease association network

Based on the lncRNA-disease association network, the lncRNA Jaccard similarity value ${JL}_{LD}\left(l_{i},l_{j}\right)$ between lncRNAs $l_{i}$ and $l_{j}$ is described as,

$${JL}_{LD}\left(l_{i},l_{j}\right)=\frac{\left|{IP}_{LD}\left(l_{i}\right)\cap {IP}_{LD}\left(l_{j}\right)\right|}{\left|{IP}_{LD}\left(l_{i}\right)\cup {IP}_{LD}\left(l_{j}\right)\right|}$$ (14)

where ${IP}_{LD}\left(l_{i}\right)$ is the vector of lncRNA $l_{i}$ in the lncRNA-disease association matrix, the same as the representation of $IP\left(l_{i}\right)$ .

#### 2.2.3 LncRNA Jaccard similarity network based on the lncRNA-miRNA association network

Likewise, lncRNA-miRNA association network is also introduced for calculating the lncRNA Jaccard similarity value ${JL}_{LM}\left(l_{i},l_{j}\right)$ between lncRNAs $l_{i}$ and $l_{j}$ , which is defined as,

$${JL}_{LM}\left(l_{i},l_{j}\right)=\frac{\left|{IP}_{LM}\left(l_{i}\right)\cap {IP}_{LM}\left(l_{j}\right)\right|}{\left|{IP}_{LM}\left(l_{i}\right)\cup {IP}_{LM}\left(l_{j}\right)\right|}$$ (15)

where ${IP}_{LM}\left(l_{i}\right)$ is the vector of lncRNA $l_{i}$ in the lncRNA-miRNA association network.

### 2.3 iLncDA-RSN

**FIGURE 1:**
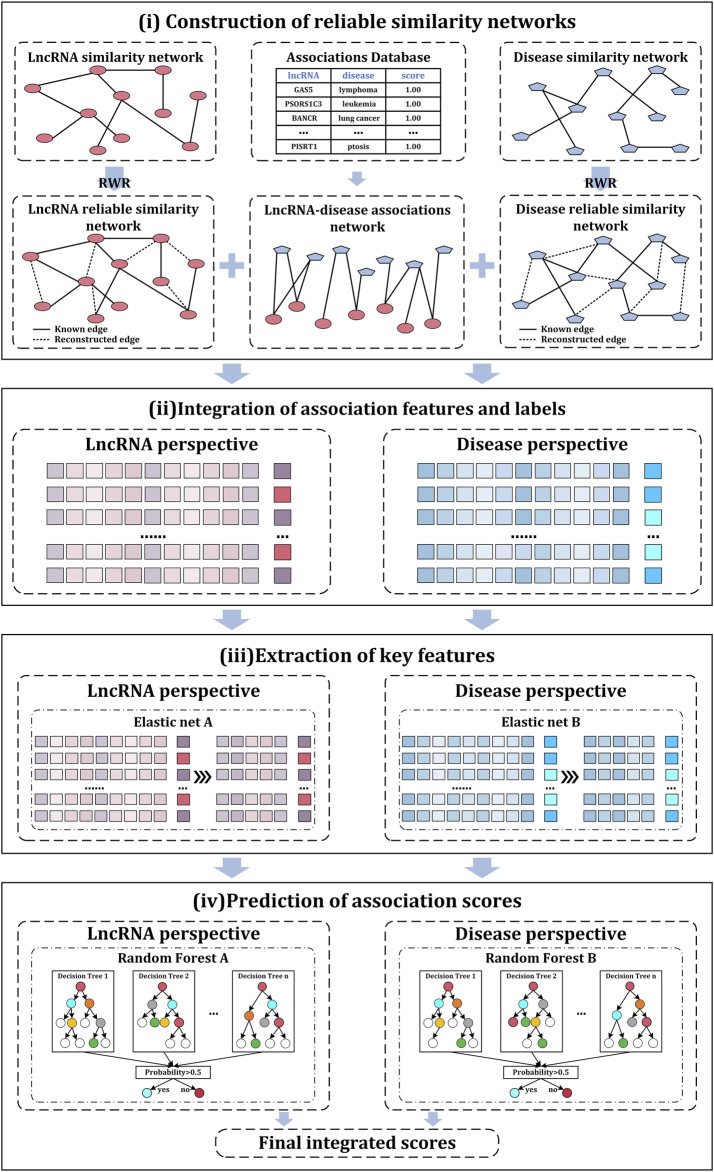
Flowchart of the iLncDA-RSN.

In this study, a computational model iLncDA-RSN is proposed for the Identification of LncRNA-Disease Associations based on Reliable Similarity Networks. Figure 1 shows its flowchart, from which it is seen that the iLncDA-RSN mainly has four steps, i.e., construction of reliable similarity networks, integration of association features and labels, extraction of key features, and prediction of association scores.

#### 2.3.1 Construction of reliable similarity networks

One type of similarity network of lncRNAs or diseases only describe their biological characteristics in a single perspective and multiple types of similarity networks of lncRNAs (or diseases) can complementary and comprehensively characterize their similarities. Hence, it is a challenge to properly integrate them without bringing in redundancy and noises. In this study, a random walk with restart (RWR) strategy is applied to construct reliable similarity networks, rather than directly fuse similarity networks together, since RWR can take into account the topological connectivity patterns globally and locally within the network by introducing predefined restart probabilities at the initial nodes of each iteration to exploit potential relationships between nodes, either directly or indirectly (Liao et al., 2009; Cao et al., 2014). Specifically, W is defined as the weighted adjacency matrix of a similarity network with $n_{d}$ diseases (or $n_{l}$ lncRNAs), T is the probability matrix where each element $T\left(i,j\right)$ represents the transition probability from node i to node j , which can be written as,

$$T\left(i,j\right)=\frac{W\left(i,j\right)}{\sum W\left(i,\cdot \right)}$$ (16)

Then, $S_{i}^{t}$ is defined as a $n_{d}$ dimensional vector, in which the probability of each node being visited after t iterations from the node i during the random walk is stored. The RWR that starts from the node i can be described as,

$$S_{i}^{t+1}=\left(1-p_{r}\right)S_{i}^{t}T+p_{r}e_{i}$$ (17)

where $e_{i}$ represents the $n_{d}$ dimensional standard basis vector, and $p_{r}$ represents the predefined restart probability, which serves to control the mutual influence of global and local topological information during diffusion, the higher value placing more emphasis on the local structure in the network. After a certain number of iterations, we can obtain the smooth distribution $S_{i}^{\infty }$ of the RWR, i.e., the diffusion state of that node, $S_{i}=S_{i}^{\infty }$ . If two nodes have similar diffusion states, it usually means that they share similar locations concerning other nodes in the network and therefore may share similar functions (Luo et al., 2017). Using the RWR strategy, the disease integrated similarity network SD , the disease Jaccard similarity networks ${JD}_{LD}$ and ${JD}_{LD}$ are constructed as the disease reliable similarity network RD . Similarly, the lncRNA integrated similarity network SL , the lncRNA Jaccard similarity networks ${JL}_{LD}$ and ${JL}_{LM}$ are constructed as the lncRNA reliable similarity network RL .

#### 2.3.2 Integration of association features and labels

Depending on the lncRNA-disease association network LD and the reliable similarity networks RD , RL , feature vectors of lncRNA-disease pairs are integrated from lncRNA and disease perspectives respectively (Liu et al., 2022). Specifically, from the disease perspective, the reliable similarity vector of each disease in RD is exhaustively combined with the lncRNA vector of each disease in LD , resulting in an association feature set of all lncRNA-disease pairs with $n_{d}\times n_{l}$ samples and $n_{d}+n_{l}$ features; from the lncRNA perspective, the reliable similarity vector of each lncRNA in RL is exhaustively combined with the disease vector of each lncRNA in LD , resulting in another association feature set of all lncRNA-disease pairs with $n_{d}\times n_{l}$ samples and $n_{d}+n_{l}$ features.

Labels of samples in these two association feature sets are marked as known LDAs, i.e., if the lncRNA-disease pair between the disease d and the lncRNA l belong to the known LDAs, its label is 1, otherwise, 0.

#### 2.3.3 Extraction of key features

To remove redundant features from the association feature sets to improve the prediction accuracy of LDAs, a feature extraction method, i.e., elastic net (Liu et al., 2020) is employed in this study. The elastic net is a regularization and variable selection method that has been widely used for processing data (Yu et al., 2021). The elastic net employs two penalty terms ( $l_{1}-norm$ and $l_{2}-norm$ ) to automatically select important features and perform continuous shrinkage to improve prediction accuracy. Suppose the feature set is $X=\left[x_{1},x_{2},\cdots ,x_{N}\right]\in R^{N\times d}$ , and its corresponding label vector is $Y=\left[y_{1},y_{2},\cdots ,y_{N}\right]\in R^{N}$ , the linear regression model and the elastic net are respective defined as,

$$\min_{\omega }{\sum_{i=1}^{N}\left(y_{i}-\omega^{T}x_{i}\right)}^{2}$$ (18)

$$\min\frac{1}{2\times N}{\left\|Y-X\omega \right\|}_{2}^{2}-\alpha \times \beta {\left\|\omega \right\|}_{1}+\frac{1}{2}\alpha \times \left(1-\beta \right){\left\|\omega \right\|}_{2}^{2}$$ (19)

where the penalty degree of the model is controlled by adjusting the weight terms α and β for variable selection.

#### 2.3.4 Prediction of association scores

The random forest is based on the idea of Bagging ensemble learning, which introduces sample randomness and attributes randomness. With strong robustness and generalization, the random forest is extensively applied in the field of bioinformatics (Chen et al., 2018; Wei et al., 2021). In this study, we also apply the random forest to the iLncDA-RSN as its classifier to predict the scores of LDAs. Since there are two lncRNA-disease association feature sets constructed from lncRNA and disease perspectives respectively, two random forests are used together on them to identify potential LDAs. The final predicted association score $Score\left(d,l\right)$ of the iLncDA-RSN between the disease d and the lncRNA l is,

$$Score\left(d,l\right)=\frac{S_{RFd}\left(d,l\right)+S_{RFl}\left(d,l\right)}{2}$$ (20)

where $S_{RFd}\left(d,l\right)$ is the random forest association score between the disease d and the lncRNA l on the lncRNA-disease association feature set from the disease perspective.

## 3 Results

In the study, a lncRNA-disease association network is downloaded from the Lnc2Cancer (Ning et al., 2016), GeneRIF (Lu et al., 2007) and LncRNADisease (Chen et al., 2013) databases, which includes 412 diseases, 240 lncRNAs, and 2,697 known LDAs. For a fair experimental comparison, we divided 80% of the samples into the benchmark dataset and the remaining 20% into the independent validation set (Zhang et al., 2022). The benchmark dataset is employed to select optimal parameters as well as to train the iLncDA-RSN, while the independent validation set is employed to compare the iLncDA-RSN with other computational models. To provide prior knowledge for accurately identifying potential LDAs, a miRNA-disease association network is introduced from the HMDD 2.0 database (Li et al., 2014), in which includes 13,562 experimentally validated miRNA-disease associations, and a lncRNA-miRNA association network is also introduced from the starBase database (Li et al., 2014), in which includes 1,002 experimentally validated lncRNA-miRNA associations.

We performed the 5-fold cross-validation on the benchmark dataset and used five evaluation metrics to evaluate the iLncDA-RSN, i.e., area under the receiver operating characteristic curve (AUC), Accuracy (Acc), Sensitivity (Sen), Matthews correlation coefficient (MCC) and F1-score (F1), which are defined as,

$$Acc=\frac{TN+TP}{TN+TP+FN+FP}$$ (21)

$$Sen=\frac{TP}{TP+FN}$$ (22)

$$MCC=\frac{TP\times TN-FP\times FN}{\sqrt{\left(TP+FN\right)\times \left(TP+FP\right)\times \left(TN+FN\right)\times \left(TN+FP\right)}}$$ (23)

$$F1=\frac{2TP}{2TP+FP+FN}$$ (24)

where TP , FN , TN , and FP represent true positives, false negatives, true negatives and false positives, respectively.

### 3.1 Evaluation of prediction ability

**FIGURE 2:**
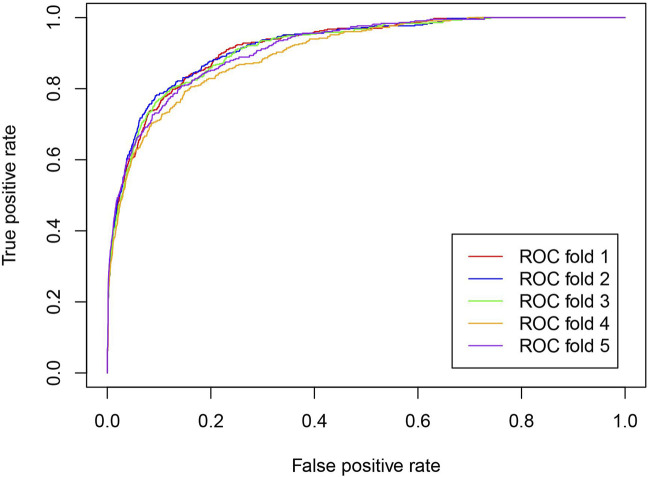
ROC curves of the iLncDA-RSN on the benchmark dataset under the 5-fold cross-validation.

To comprehensively evaluate the prediction ability of the iLncDA-RSN, this study performed experiments on the benchmark dataset using the 5-fold cross-validation, and evaluated experimental results using 5 metrics, including AUC, Acc, Sen, MCC, and F1. Table 1 lists its experimental results, from which it is seen that the iLncDA-RSN obtained an average AUC of 91.59%, Acc of 90.70%, Sen of 91.36%, MCC of 81.34% and F1 of 90.75%, respectively. These results demonstrate that the iLncDA-RSN has high prediction ability and can play an important role in identifying potential LDAs. Besides, it is also seen that the prediction ability of the iLncDA-RSN is stable since the standard deviations are small in terms of 5 metrics. Figure 2 shows receiver operating characteristic (ROC) curves of the iLncDA-RSN on the benchmark dataset under the 5-fold cross-validation. It is seen that the ROC curves on different test sets are very similar, implying that its high stability and reliability.

**TABLE 1:** 5-Fold cross-validation results of the iLncRNA-RSN on benchmark dataset.

Test set	Acc (%)	Sen (%)	Mcc (%)	F1 (%)	AUC (%)
1	91.73	91.62	81.94	91.78	92.05
2	92.16	90.75	82.51	91.03	92.23
3	91.12	92.84	82.75	90.56	91.76
4	88.45	89.86	76.51	89.01	90.32
5	90.04	91.71	83.00	91.38	91.59
Average	90.70 ± 1.49	91.36 ± 1.12	81.34 ± 2.73	90.75 ± 1.07	91.59 ± 0.75

### 3.2 Evaluation of the reliable similarity network

**FIGURE 3:**
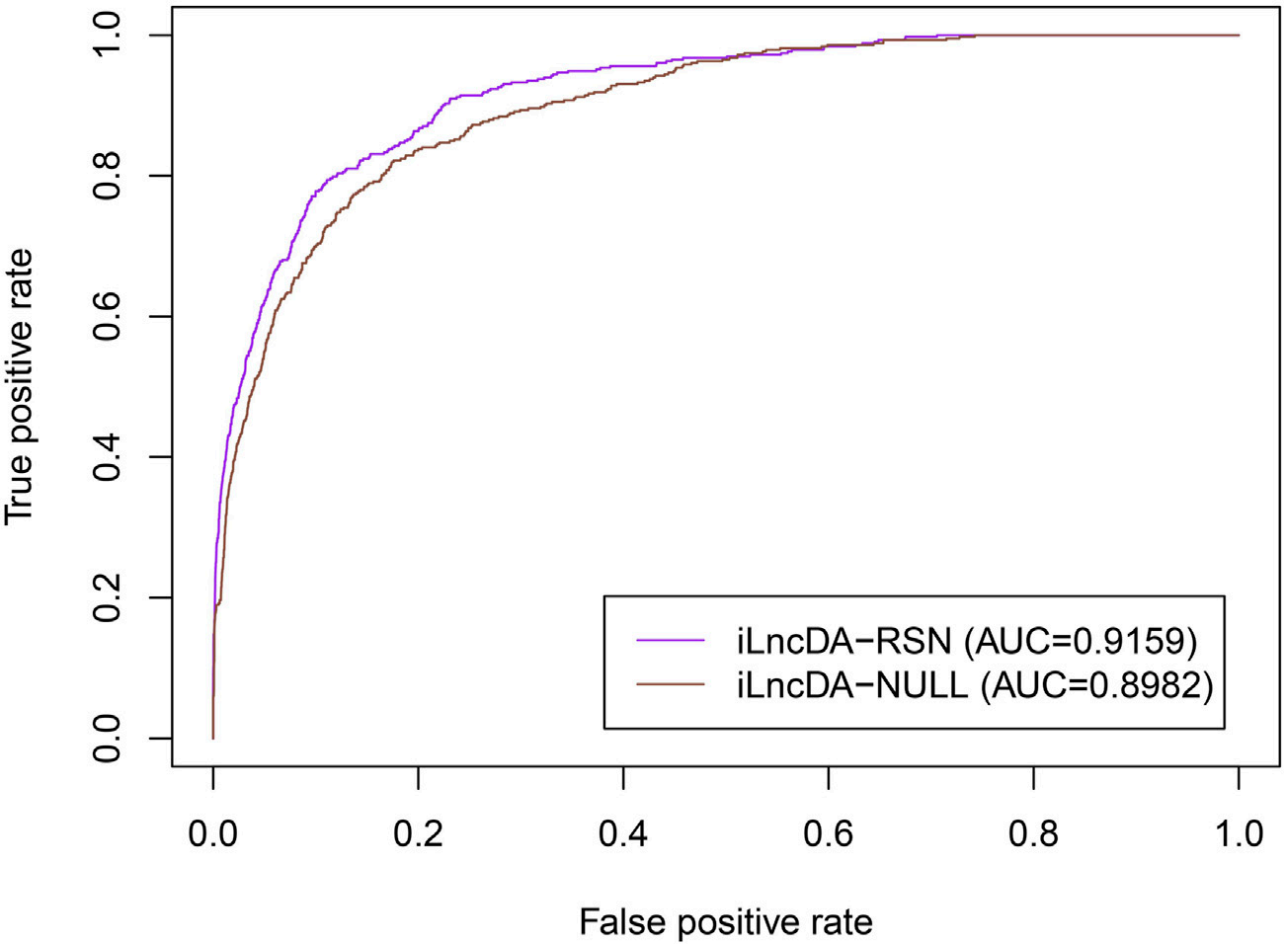
ROC curves of the iLncDA-RSN and the iLncDA-NULL on the benchmark dataset.

To demonstrate that the reliable similarity network is important for the iLncDA-RSN to improve the prediction ability, we performed a comparison experiment between the iLncDA-RSN and the iLncDA-NULL. Compared with the iLncDA-RSN, the iLncDA-NULL uses the directly integrated similarity networks of lncRNAs and diseases, rather than reliable similarity networks. For a fair comparison, all experimental steps and parameter settings are the same. Figure 3 shows ROC curves of the iLncDA-RSN and the iLncDA-NULL under the 5-fold cross-validation on the benchmark dataset. It is seen that the iLncDA-RSN significantly outperforms the iLncDA-NULL with their respective AUC values being 0.9159 and 0.8982, implying that the reliable similarity network is indeed important for improving the prediction ability.

### 3.3 Evaluation of the miRNA heuristic information

**FIGURE 4:**
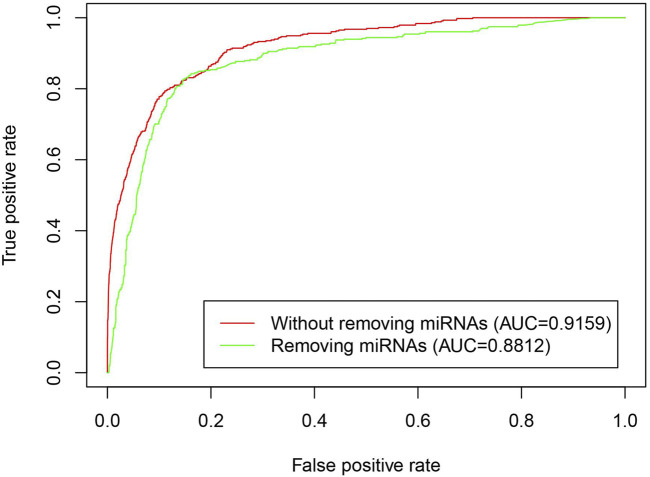
ROC curves of the iLncDA-RSN with and without miRNA heuristic information on the benchmark dataset.

To validate that the iLncDA-RSN is advantageous by introducing the miRNA heuristic information to construct reliable similarity network, we performed a comparison experiment between the iLncDA-RSN and the same model that does not introduce the miRNA heuristic information. Figure 4 shows ROC curves of the iLncDA-RSN with and without miRNA heuristic information on the benchmark dataset. It is seen that the iLncDA-RSN is significantly superior to the model without introducing the miRNA heuristic information in terms of AUC, implying that the introduced miRNA heuristic information can help to provide supplementary prior knowledge for accurately identifying potential LDAs.

### 3.4 Comparison with other dimensionality reduction methods

**FIGURE 5:**
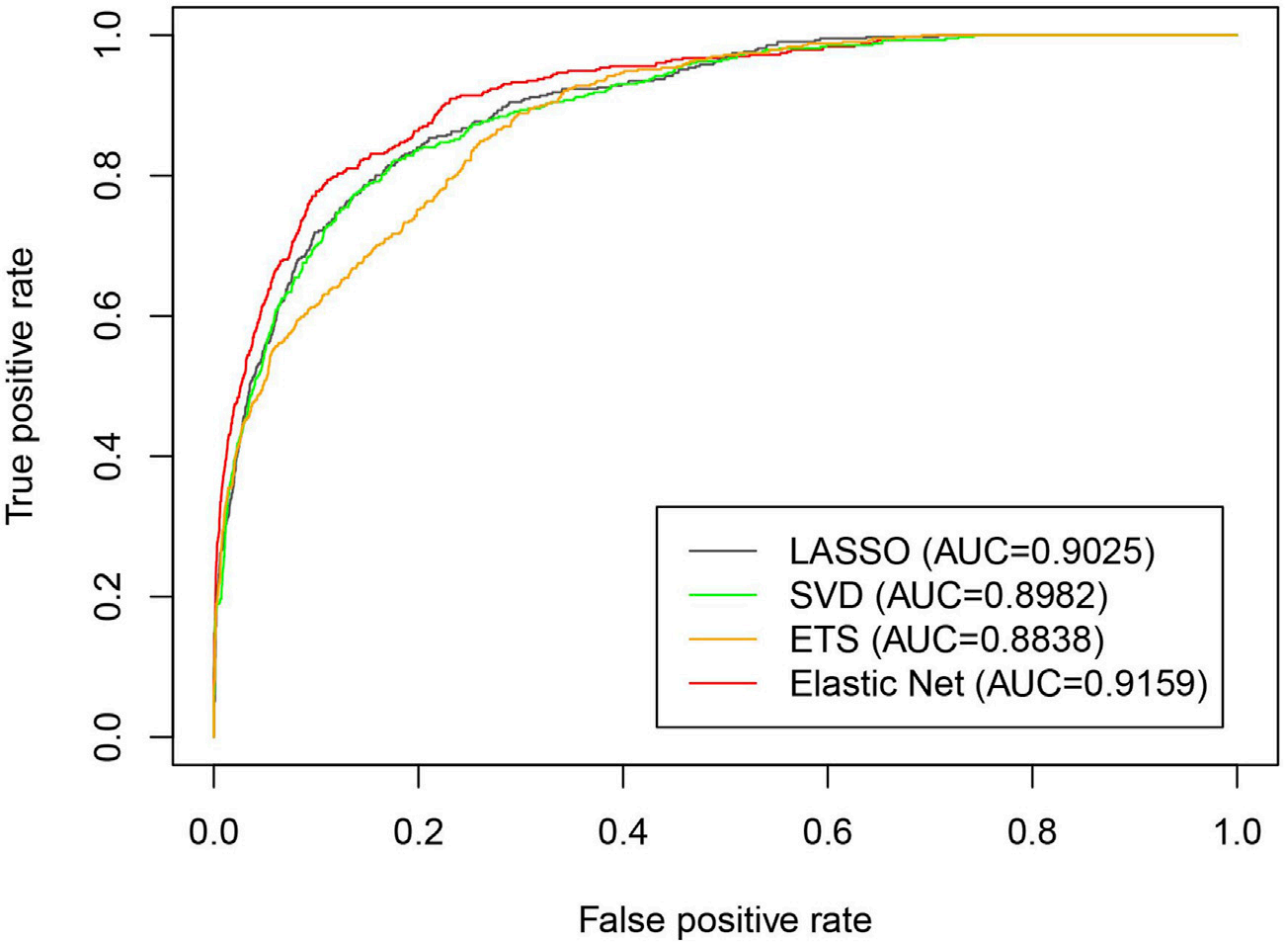
ROC curves of the iLncDA-RSN with different dimensionality reduction methods on the benchmark dataset.

To test the performance of the elastic net for dimensionality reduction in the iLncDA-RSN, we compared it with other three dimensionality reduction methods, including extra-trees (ETS) (Liu et al., 2020), LASSO (Ranstam and Cook, 2018) and SVD (Zeng et al., 2020). The feature extraction part of the iLncDA-RSN is replaced by these three dimensionality reduction methods and other parts are the same to ensure a fair comparison. Figure 5 shows ROC curves of the iLncDA-RSN with different dimensionality reduction methods on the benchmark dataset. It is seen that their AUC values are 0.9025, 0.8982, 0.8838, and 0.9159 corresponding to LASSO, SVD, ETS and the elastic net, respectively. Hence, in the iLncDA-RSN, the elastic net method is employed to remove redundant features from the association feature sets to improve the prediction accuracy of LDAs.

### 3.5 Comparison with other classifiers

**FIGURE 6:**
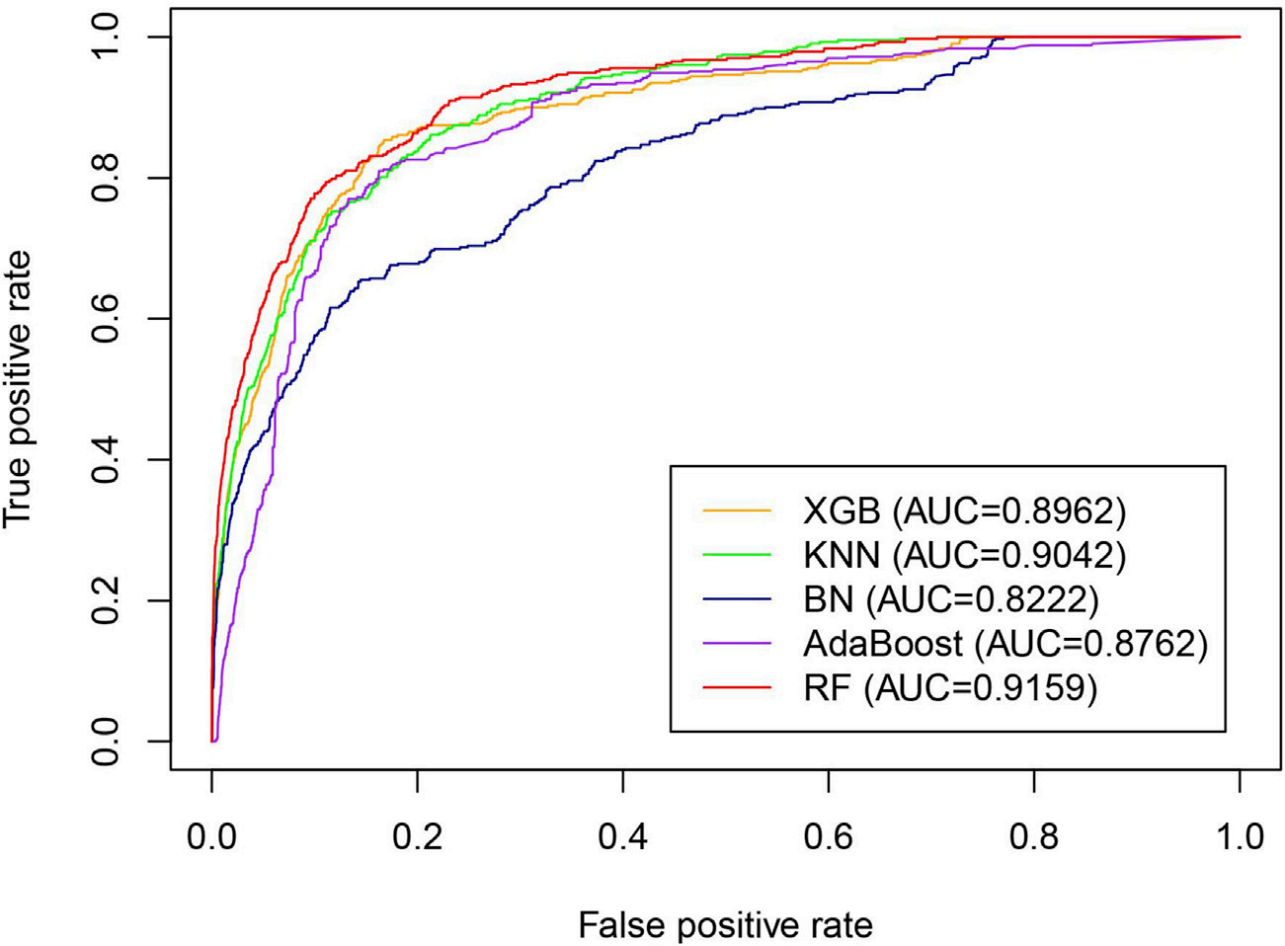
ROC curves of the iLncDA-RSN with different classifiers on the benchmark dataset.

To find the most suitable classifier for the iLncDA-RSN, multiple classic classifiers, including random forest (RF), XGBoost (XGB) (Chen and Guestrin, 2016), k-nearest neighbor (KNN) (Liu et al., 2020), AdaBoost (Zhao et al., 2019) and Bayesian network (BN) (Marcot and Penman, 2019), were tested. Figure 6 shows ROC curves of the iLncDA-RSN with different classifiers on the benchmark dataset. It is seen that AUC values of RF, XGB, KNN, AdaBoost, and BN are 0.9159, 0.8962, 0.9042, 0.8762, and 0.8222, respectively, implying that the winner random forest is the most suitable classifier among them.

### 3.6 Comparison with other computational models

**FIGURE 7:**
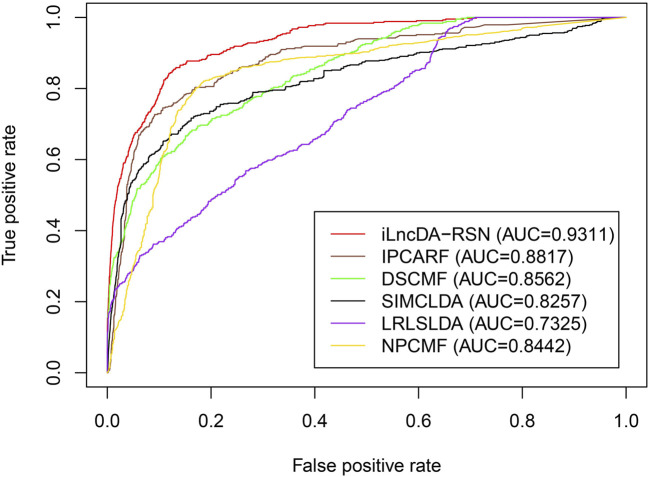
ROC curves of compared computational models on the independent validation set.

To further evaluate the prediction ability of the iLncDA-RSN, 5-fold cross-validation was performed to compare the iLncDA-RSN and other five state-of-the-art models, including IPCARF (Zhu et al., 2021), DSCMF (Liu et al., 2021), SIMCLDA (Lu et al., 2018), LRLSLDA (Chen and Yan, 2013) and NPCMF (Gao et al., 2019) on the independent validation set. Figure 7 shows ROC curves of all compared computational models. It is seen that the iLncDA-RSN has the largest area under the ROC curve, achieving an AUC value of 0.9311, while the other five computational models have AUC values of 0.8817, 0.8562, 0.8257, 0.7325, and 0.8442, respectively. This indicates that the iLncDA-RSN has better prediction ability and can predict potential LDAs more accurately.

### 3.7 Case study

To validate the ability of the iLncDA-RSN in predicting potential LDAs, we performed case studies for cervical cancer, colon cancer and gastric cancer. All known LDAs and miRNA-disease associations were employed to train the iLncDA-RSN, which then predicts lncRNAs associated with each disease, and gives their association scores. The predicted lncRNAs were ranked based on their association scores and the top 15 lncRNAs would be verified through the databases Lnc2Cancer v2.0 (Ning et al., 2016) and lncRNADisease v2.0 (Chen et al., 2013).

Cervical cancer is diagnosed in more than 500,000 women, which causes more than 300,000 deaths worldwide (Jiang et al., 2021). Top 15 lncRNAs predicted by the iLncRNA-RSN for the cervical cancer is recorded in Table 2. Through a series of experiments, Zhang et al. (2017) demonstrated that the expression of lncRNA CDKN2B-AS1 is remarkably high in both cervical cancer tissues and cell lines, and the CDKN2B-AS1 may take an essential part in the progression of cervical cancer, implying that CDKN2B-AS1 may work as a new cervical cancer therapeutic target and prognostic biomarker. Wang and Zhu (2018) demonstrated that lncRNA NEAT1 serves as a miR-101 sponge in cervical cancer and its upregulated level is associated with poor prognosis and poor clinical-pathological factors, implying that NEAT1 might be a target for the treatment of cervical cancer. Yan et al. (2018) performed a luciferase reporter gene analysis, which showed that there is a binding site between the UCA1 lncRNA and miR-206, and the UCA1 is upregulated in the tissues of cervical cancer patients.

**TABLE 2:** Top 15 lncRNAs predicted by the iLncRNA-RSN for the cervical cancer.

Rank	LncRNA	Evidence
1	CDKN2B-AS1	C^a^&D^b^
2	NEAT1	C&D
3	CDKN2A-AS1	D
4	MIR17HG	D
5	UCA1	C&D
6	KCNQ1OT1	D
7	HCP5	C&D
8	TP53COR1	C&D
9	MIR155HG	D
10	HOTTIP	D
11	DANCR	C&D
12	XIST	C&D
13	ATXN8OS	D
14	TP53TG1	D
15	LINC00299	D

^a^ C represents Lnc2Cancer v2.0 database.

^b^ D represents lncRNADisease v2.0 database.

Colon cancer, a common preventable cancer, has been increasing in incidence and mortality among young people under the age of 50 in the past 25 years (Ahmed, 2020). Top 15 lncRNAs predicted by the iLncRNA-RSN for the colon cancer is recorded in Table 3. Of them, 14 lncRNAs are verified in databases C and D. (Tseng et al., 2014) found that lncRNA PVT1 increases MYC protein level, which in turn increases the cancer rate of colon cancer. (Li et al., 2019) showed that lncRNA KCNQ1OT1 fosters chemoresistance in colon cancer via sponging miR-34a and may act as a possible target for the therapy of colon cancer. (Sun et al., 2018) used qRT-PCR to measure the expression of lncRNA XIST in colon cancer tissues as well as in adjacent normal tissues, and showed that XIST expression is upregulated remarkably in tissues of colon cancer, thus indicating that XIST plays an oncogenic role in colon cancer.

**TABLE 3:** Top 15 lncRNAs predicted by the iLncRNA-RSN for the colon cancer.

Rank	LncRNA	Evidence
1	CDKN2B-AS1	C&D
2	GAS5	C&D
3	MIR17HG	D
4	PVT1	C&D
5	DISC2	D
6	KCNQ1OT1	C&D
7	NEAT1	C&D
8	XIST	C&D
9	HCP5	Unidentified
10	ATXN8OS	D
11	PISRT1	D
12	MIAT	D
13	MIR155HG	C&D
14	UCA1	C&D
15	SPRY4-IT1	D

Most patients with gastric cancer are diagnosed at an advanced phase and suffer from a poor prognosis (Lian et al., 2016). Top 15 lncRNAs predicted by the iLncRNA-RSN for the gastric cancer is recorded in Table 4. Several studies (Chang et al., 2016; Wang et al., 2016; Ye et al., 2016) found that lncRNA HOTTIP may play a significant part in the initiation and progression of gastric cancer, and may be both a new prognostic marker and a prospective target for the therapy of gastric cancer. Sha et al. (2018) conducted real-time PCR with gastric cancer specimens and adjacent matched regular tissues, and showed that the level of lncRNA MIAT in gastric cancer tissues is elevated. (Tan et al., 2019b) found that the downregulation of lncRNA NEAT1 significantly inhibited gastric cancer progression, while overexpression of NEAT1 induced gastric cancer development. (Du et al., 2016) showed that the expression of lncRNA WT1-AS is downregulated in the tissues and cells of gastric cancer, and demonstrated that WT1-AS may be associated with gastric cancer of tumor progression.

**TABLE 4:** Top 15 lncRNAs predicted by the iLncRNA-RSN for the gastric cancer.

Rank	LncRNA	Evidence
1	ERICH1-AS1	Unidentified
2	LINC01628	D
3	NALT1	C&D
4	PISRT1	D
5	MIR100HG	C&D
6	HOTTIP	C&D
7	ATXN8OS	D
8	MIAT	C&D
9	HCP5	Unidentified
10	ESRG	D
11	MIR17HG	D
12	NEAT1	C&D
13	IFNG-AS1	D
14	LINC01080	D
15	WT1-AS	C&D

## 4 Conclusion

In this study, we presented a computational model iLncDA-RSN based on reliable similarity networks for identifying potential LDAs. Specifically, for constructing reliable similarity networks of lncRNAs and diseases, miRNA heuristic information with lncRNAs and diseases is firstly introduced to construct their respective Jaccard similarity networks; then GIP kernel similarity networks and Jaccard similarity networks of lncRNAs and diseases are provided based on the lncRNA-disease association network; a random walk with restart strategy is finally applied on Jaccard similarity networks, GIP kernel similarity networks, as well as lncRNA functional similarity network and disease semantic similarity network to construct reliable similarity networks. Depending on the lncRNA-disease association network and the reliable similarity networks, feature vectors of lncRNA-disease pairs are integrated from lncRNA and disease perspectives respectively, and then dimensionality reduced by the elastic net. Two random forests are at last used together on different lncRNA-disease association feature sets to identify potential LDAs. The iLncDA-RSN is evaluated by five-fold cross-validation and five experiments were performed, including evaluation of prediction ability, evaluation of the reliable similarity network, evaluation of the miRNA heuristic information, comparison with other dimensionality reduction methods, comparison with other classifiers, and comparison with other computational models. Experimental results show that the iLncDA-RSN outperforms the compared models. Furthermore, case studies of different complex diseases demonstrate the effectiveness of the iLncDA-RSN in identifying potential LDAs.

## Data Availability

The original contributions presented in the study are included in the article/Supplementary Material, further inquiries can be directed to the corresponding author.
